# A total evidence approach justifies taxonomic splitting of the endangered Pecos gambusia into three species

**DOI:** 10.1098/rsos.251025

**Published:** 2025-11-26

**Authors:** David S. Portnoy, Robert J. Bretzing-Tungate, Andrew T. Fields, Megan G. Bean, Ryan K. Smith, Elizabeth P. Dolan, Rose Blanchard, Kevin W. Conway

**Affiliations:** ^1^ Department of Life Sciences, Texas A&M University—Corpus Christi, Corpus Christi, TX, USA; ^2^ Austin Ecological Services Field Office, US Fish and Wildlife Service, Austin, TX, USA; ^3^ The Nature Conservancy, San Antonio, TX, USA; ^4^ Department of Natural Sciences, Northwest Missouri State University, Maryville, MO, USA; ^5^ Department of Ecology and Conservation Biology, Texas A&M, College Station, TX, USA

**Keywords:** Chihuahua desert, genetic drift, taxonomy, Poeciliidae, species delimitation

## Abstract

*Gambusia nobilis* is a federally endangered species found across a fragmented distribution within the Pecos River Drainage of Texas and New Mexico, USA. Drought, human water usage, and potential hybridization and competition with introduced congeners threaten species persistence. Therefore, a population genomics study was conducted to provide critical information for conservation planning. Unsupervised clustering suggested hierarchical structure, with a primary *K* = 3, and deep divergences were detected among samples grouped into the Leon Creek watershed, the Toyah Creek watershed, and water bodies within the Bitter Lake National Wildlife Refuge (*F’*_ST_ = 0.55–0.76 for putatively neutral data). Phylogenetic analyses showed three distinct clades corresponding to these groups, with divergence times estimated to be in the last 50 000 years. Complimentary morphological analyses detected differences among the three groups, including features of male colour pattern, and the number of caudal-fin rays in both sexes. Taken as a whole, the results indicate that the endangered *G. nobilis* comprises three species (two of which are named herein as *G. pyrros* n. sp. and *G. echelleorum* n. sp.), rather than one, and the study highlights the daunting yet critical task of documenting species diversity during a period of unprecedented diversity loss.

## 1. Introduction

Currently, the Earth is in the midst of a mass extinction event which differs from the previous five known events in terms of the relative rate of diversity loss and the role of human activity as the primary driver [1,2]. The importance of anthropogenic impacts on basic properties of the Earth has led some to propose a unique geological epoch, referred to as the Anthropocene [3], and the associated diversity crisis involves not only elevated extinction rates, but the homogenization of diversity across broad geographic expanses through extirpation, species introductions and novel anthropogenic selective pressures [4,5]. At the same time, technological advances in genetic techniques have led to an increased ability to detect cryptic diversity within and between species [6], posing a challenge to conservationists as they are charged with documenting and protecting diversity while acutely aware that they do not fully understand its scope. This creates a potential ‘Achilles and the tortoise’ dynamic, where conservation efforts lag several steps behind, making studies aimed at describing diversity using a variety of complementary approaches (e.g. genetic, morphological, ecological) critical.

The problem of anthropogenically driven diversity loss, occurring over the background of undescribed diversity is particularly acute in freshwater systems. Freshwater systems are particularly vulnerable to anthropogenic stressors due to their importance, and by design, proximity to human civilization [7]. Habitat alterations via damming, landscape alteration (e.g. logging, urbanization) and diversions have occurred over many hundreds of years [8] and are compounded more recently by increased water demand exacerbated by climate change, unsustainable fisheries practice and widespread species introductions [9]. The resulting pressure on freshwater systems has led to extinction rates forecasted to outpace those seen in terrestrial systems, with close to one quarter of freshwater species currently estimated to be threatened with extinction [10]. Because freshwater systems tend to be closed (i.e. bounded by land and marine systems), there is opportunity for diversification at small geographic scales over short periods of geological time [11], especially for opportunistic species with short generation times. Therefore, the recent description of freshwater species has not been limited to taxa found in remote locations but often involves the close examination (genetic and morphological) of widespread species with disjunct or fragmented distributions [12].

Many freshwater fishes of the southwestern United States occupy fragmented habitats across their distribution due to natural water limitation and extreme environmental fluctuations, including periods of hypersalinity and hypoxia due to seasonal drought and temperature [13]. These species are thought to have dispersed during past climate regimes that featured increased precipitation and decreased evaporation in the region [14,15], particularly during monsoon regimes during the Pliocene (3.5–2.5 ma [16]). As conditions in the region became more arid, many species became restricted to small, isolated habitats fed by groundwater, where they persist as a series of isolated populations [15]. Spring-fed freshwater sources are also vital for human activities in arid desert landscapes, bringing species in the region into proximity of ranches, oilfields and population centres. This creates a delicate situation in which building anthropogenic pressures on naturally limited habitat is exacerbating vulnerability of species, making regular population monitoring, and in some cases maintenance of captive reserve populations, necessary to prevent extirpation and extinction [17].

The focal species of this study, the Pecos gambusia (*Gambusia nobilis*), inhabits spring-associated habitats of the Pecos River Drainage in Texas and New Mexico, USA [18,19]. The species has been extirpated from much of its natural range and is federally listed as endangered. The current remaining populations are found in several disjunct, seemingly isolated geographic groups and contemporary threats to species persistence include predation by introduced species, increasingly common drought conditions, human water usage and hybridization and competition with the introduced congeneric *G. affinis* and *G. geiseri* [20]. Previous work based on allozymes documented genetic differentiation among regionally clustered populations [19], but a follow-up study using modern genomic techniques was warranted to provide data for conservation planning, including a better understanding of the scope of potential hybridization and introgression, level of divergence among populations, and the amount of standing genetic variation within and across those populations. The results described here showed surprisingly deep levels of divergence among the three regions. Subsequent phylogenetic and morphological assessment confirmed the presence of three species rather than one, including two new species described herein.

## 2. Methods

### 2.1. Sample collection

**Figure 1.**
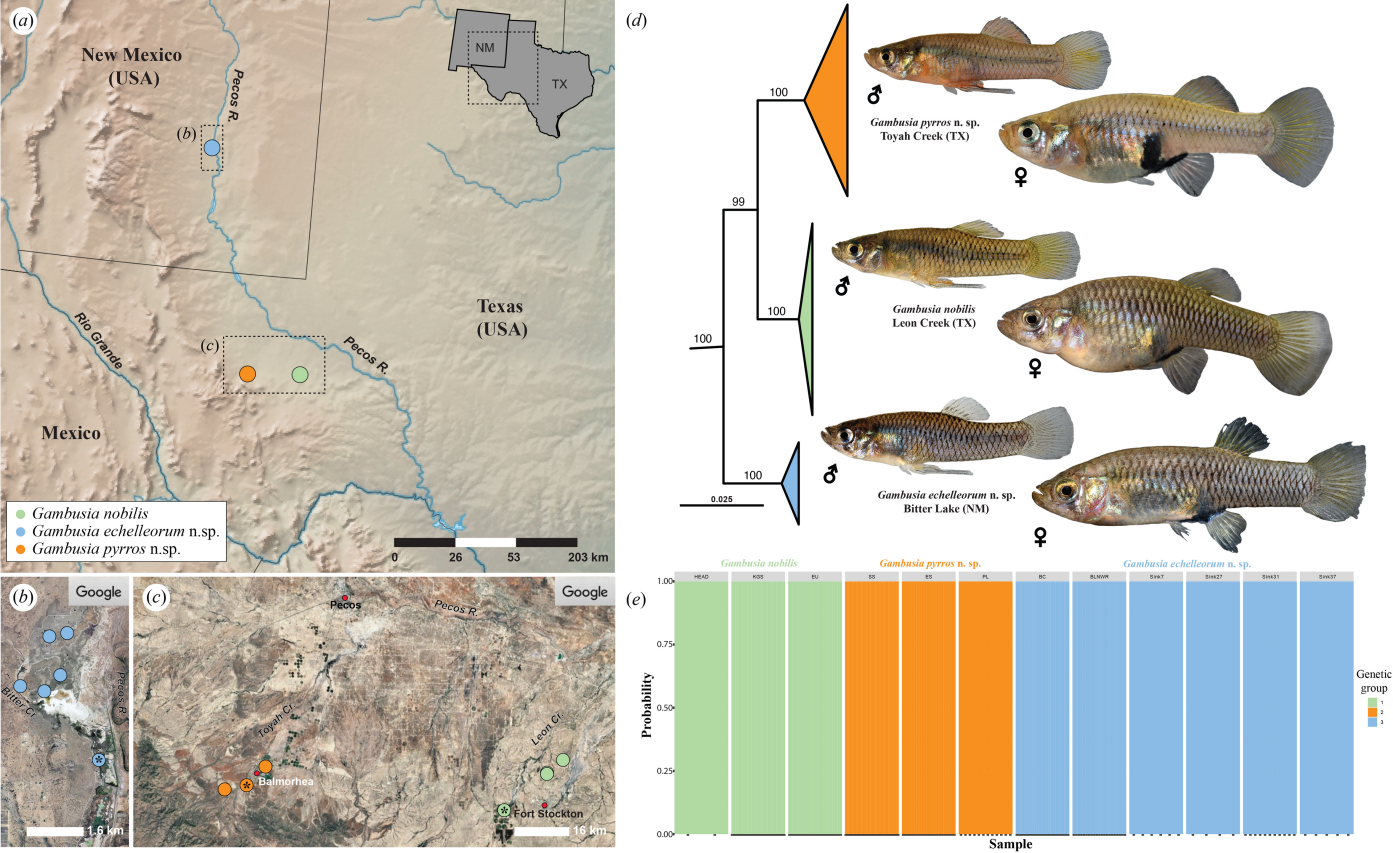
Distribution and relationships of the *Gambusia nobilis* species complex. (*a*) Map showing distribution of *G. nobilis*, *G. pyrros* n. sp. and *G. echelleorum* n.sp. within Chihuahuan desert ecoregion of western Texas and southeastern New Mexico. (*b*) Closer view of area surrounded by dashed rectangle (*b*) in (*a*), showing location of *G. echelleorum* samples from Bitter Lake watershed in New Mexio, type locality indicated by black asterisk (*). (*c*) Closer view of area surrounded by dashed rectangle (*c*) in (*a*), showing location of *G. nobilis* samples from Leon Creek watershed and *G. pyrros* samples from Toyah Creek watershed in Texas, type localities indicated by black asterisk (*). (*d*) Clade equivalent to the *Gambusia nobilis* species complex from the Maximum Likelihood phylogram based on 5989 loci showing relationships of *G. nobilis*, *G. pyrros* and *G. echelleorum*, numbers above branches represent bootstrap values (full topology available in electronic supplementary material, figure S7). (*e*) Discriminant analysis of principal components using the unsupervised clustering algorithm, *K*-means (= 3), using 3502 single nucleotide polymorphism-containing loci and 212 individuals (*G. nobilis*, *n* = 63; *G. pyrros*, *n* = 79; *G. echelleorum*, *n* = 70). Basemap in (*a*) created with SimpleMappr. Satellite images in (*b*) and (*c*) obtained from Google Earth.

Fin clips or voucher specimens of *Gambusia nobilis* were collected between 2020 and 2024, from 12 discrete sampling sites within the Pecos River drainage (a sub-basin of the larger Rio Grande drainage) of Texas and New Mexico. This included three springs (Diamond Y Head Pool, HEAD; Karges, KGS; and Euphrasia, EU) within the Leon Creek watershed (LC) in Pecos County, Texas; three springs (San Solomon, SS; Phantom Lake, PL; and East Sandia, ES) within the Toyah Creek watershed (TC) in Jeff Davis and Reeves counties, Texas; and six sites within the Bitter Lake watershed and a series of geographically proximate sinkholes in greater Chaves County, New Mexico (NM). Sites in LC are not directly connected, though HEAD and KGS are only separated by approximately 0.3 km and connected intermittently. Sites in TC are not connected and separated by an average of 8.9 km. Sites in New Mexico included the Bitter Lake National Wildlife Refuge (BLNWR), a section of Bitter Creek north of the refuge (BC), and four sinkhole habitats (Sink7, Sink27, Sink31, Sink37), all of which are not directly connected and separated by an average of 2.0 km (figure 1; electronic supplementary material, table S1). Tissues were also collected from *G. affinis* and *G. geiseri*, when the species were encountered, but also from additional sites outside of the distribution of *G. nobilis* (electronic supplementary material, table S1). Additional tissues were acquired from four other species of *Gambusia* found in Texas (*G. heterochir*, *G. clarkhubbsi*, *G. speciosa*, *G. gagei*) and from other species in the family Poeciliidae (electronic supplementary material, table S1). Metadata and voucher numbers can be found in electronic supplementary material, S1.

### 2.2. Sequencing and data processing

DNA was extracted using Mag-Bind Tissue DNA kits (Omega Bio-Tek) and approximately 1000 ng of high-quality genomic DNA was used in a modified version of the ddRAD genomic library preparation method [21]. Libraries were sequenced on part of an Illumina NovaSeq X lane with technical replicates (duplicated individuals) sequenced across the libraries. In total, three libraries were sequenced with 356 unique individuals.

Raw reads were demutliplexed in the software Stacks [22]. Read trimming, mapping and SNP calling were performed using dDocent [23]. After trimming with *fastp* v.0.23.2 [24], overlapping reads were concatenated with *pear* v.0.9.6 [25] before mapping both overlapping reads and nonoverlapping reads to the *Gambusia affinis* genome (GenBank no. GCF_019740435). Individual SNPs were identified and compiled into a variant call file (VCF) file using *freebayes* v.1.0.2 [26] and variants were filtered using a combination of *VCFtools* v.0.1.17 [27] and custom BASH and Perl scripts to remove artefacts [28]. SNPs on the same RAD fragment were collapsed into microhaplotypes (SNP-containing-loci) with *rad_haplotyper* v.1.1.9 and loci with more haplotypes than expected per individual were removed [29]. One individual from each pair of technical replicates were removed. Admixed individuals between *G. nobilis* and *G. geiseri* and between *G. nobilis* and *G. affinis* were identified using the Bayesian framework implemented in NewHybrids [30] and a combination of simulation and principal component analysis (PCA) and subsequently removed from the dataset.

### 2.3. Genetic structuring analysis

Population genetic analyses were conducted on a dataset that included only pure (not admixed) *Gambusia nobilis*. Relatedness was calculated, by site, using the Wang estimator [31] and confirmed using *CKMRsim* v.0.1.2.999 [32]. As related individuals are a characteristic of small populations and removing them can create bias, related individuals were not removed [33]. Two complementary methods, BayeScan v.2.1 [34] and Outflank [35], were used to identify loci potentially under selection among the three regions but also among sites within each region. Since loci under selection are not in mutation/drift equilibrium and may provide misleading demographic histories [36], all identified outlier loci were placed in separate datasets, resulting in three datasets: neutral, directional selection and balancing selection.

To identify the number of genetically distinct groups and assess for hierarchical structure, Discriminant Analysis of Principal Components [37] (DAPC) was implemented using *K*-means clustering (*k* = 1–20) [38] for all three datasets*,* with the optimal number of clusters identified by comparing Bayesian information criterion (BIC) values. Homogeneity in allele distributions among locations was tested using hierarchical locus-by-locus analysis of molecular variance (AMOVA), implemented in Arlequin v.3.5 [39], with geographic samples grouped by region, LC, TC and NM. Pairwise *F*_ST_ was subsequently estimated between all sites as well as the regions using Arlequin, with significance corrected for multiple comparisons [40]. Because background diversity can limit the magnitude of *F*_ST_ [41], pairwise estimates between regions based on the neutral dataset were corrected to *F*’_ST_.

For the neutral dataset only, mean allelic richness (*A*_r_) was estimated using *hierfstat* v. 0.5-7 in R [42] and mean expected heterozygosity (*H*_e_) was estimated using Arlequin, for each site. Homogeneity of both diversity estimates among sites was tested using Friedman’s rank sum tests [43], and post hoc Wilcoxon’s signed-rank tests [44] were used to assess pairwise differences. Contemporary effective population size (*N*_E_) was estimated for each site with a sample size of 10 or greater, using the linkage disequilibrium approach [45].

### 2.4. Phylogenetic and genetic demographic analysis

Phylogenetic analysis included a subset of *Gambusia nobilis* individuals from each region and included six additional species of *Gambusia*, as well as four outgroup taxa. A single SNP was randomly selected across each locus to avoid linkage effects. Maximum likelihood (ML) analysis was conducted in IQ-Tree v.2.2.6 [46] and net divergence between species and divergence within species were estimated as *p*-distance using MEGA v.11 [47].

BPP v.4.1.3 [48] was used to estimate the historical demographic parameters, ancestral population size (*θ*) and split time (*τ*), of *Gambusia nobilis.* Parameters were scaled to time units using the average teleost mutation rate of 5.97 × 10^–9^ per generation (range: 3.08 × 10^–9^ to 8.62 × 10^–9^; [49]) and a generation time of 1.0 year (range: 0.5 to 1.5 year [50]). Parameters generated were also used to generate the genealogical divergence index (gdi), a continuous measure that can be used to understand whether levels of divergence suggest populations within species (<0.2) or distinct species (>0.7), with ambiguity between those values due to the speciation process [51].

### 2.5. Morphological examination

Select quantitative and qualitative morphological traits were assessed in individuals of *Gambusia nobilis* representative of the three geographic groups (LC, TC and NM). Upon collection and prior to tissue subsampling, representatives of each of the groups were placed into a small field aquarium and photographed using a Nikon D850 to document life colours. Subsequent to tissue subsampling, photographed individuals were euthanized using a lethal dose of Eugenol and fixed in 10% neutral buffered formalin for a minimum of 5 days before transfer to 70% ETOH. The preserved specimens have been deposited within the Collection of Fishes at the Texas A&M University Biodiversity Research and Teaching Collections (TCWC). Approximately 10 preserved male and 10 preserved female individuals per geographic group (LC, TC and NM) were photographed in lateral view using a Zeiss SteReo Discovery V20 stereomicroscope equipped with a Zeiss Axiocam MRc5 digital camera. Fifteen measurements [52] were obtained directly from digital photographs using Fiji [53]. Counts of scales and fin rays listed in species descriptions generally follow Greenfield [54], except that the total number of caudal-fin rays (principal plus procurrent) are also reported. Terminology of the gonopodium follows Hubbs & Springer [18]. Select specimens representative of each of the three regions (LC, TC and NM) were cleared and double stained (C&S) [55]. Preserved specimens housed at the Museum of Comparative Zoology, Harvard (MCZ), Cornell Museum of Vertebrates (CU), and the University of Texas Biodiversity Center (TNHC) were also examined (see electronic supplementary material, extended methods).

To assess differences in body shape among the three geographic groups (LC, TC and NM), the position of 10 homologous landmarks visible in lateral view [56] was compared using geometric morphometrics. Homologous landmarks were placed on images of preserved specimens (approx. 10 male/10 female per TC, LC and NM) using TpsDig [57]. Following landmark placement, raw coordinate data was imported into R for analysis. To avoid issues relating to sexual dimorphism, two separate datasets were created, one for each sex (male/female). Procrustes superimposition was conducted on each coordinate dataset to translate, rotate and scale landmarks using the *geomorph* package [58,59] in R. To visualize differences in body shape between individuals of the three groups, a principal component analysis was run with the aligned coordinates for each dataset (male/female) independently using the ‘rda’ function of the *vegan* package [60] in R. PERMANOVA was conducted on the data returned for the first three principal components (PC1–3) for each of the two datasets (male/female) to assess whether significant differences in body shape existed between individuals from the three geographic groups.

More detailed descriptions of methods for all analyses can be found in electronic supplementary material, extended methods.

## 3. Results

### 3.1. Genetic structuring analysis

The final filtered dataset included 267 individuals collected from within the range of *Gambusia nobilis sensu lato* and 45 individuals collected from outside the range (*G. affinis*, *n* = 30; and *G. geiseri*, *n* = 15) genotyped at 26 156 SNP across 3505 loci. *Gambusia affinis* was found cohabitating with *G. nobilis* in LC at all three sites, with one individual identified as a *G. affinis* backcross at HEAD and two individuals identified as *G. nobilis* backcrosses at KS. In TC, *G. geiseri* was found cohabitating with *G. nobilis* in all three locations, with one individual identified as a *G. nobilis* backcross in SS and two individuals identified as *G. geiseri* backcrosses in ES. At the time of sampling in 2024, Phantom Spring had receded into the mouth of the cave and only a small number of highly inbred individuals were present, with five individuals showing evidence of admixture between *G. nobilis* and *G. geiseri*. In NM, *G. affinis* was found in BLNWR cohabitating with *G. nobilis,* and one individual was identified as an F1 hybrid between *G. nobilis* and *G. affinis*. The results of the simulation-based analysis and the NewHybrids analysis were congruent.

After removing admixed individuals the dataset included 212 individuals (LC = 63, TC = 79, NM = 70) genotyped at 12 705 SNPS across 3767 loci. The dataset included a pair of related individuals in KGS and two pairs in PHL (collected in 2022). A total of 193 loci were found to potentially be under directional selection including 192 between regions, identified by BayeScan, and one within LC identified by Outflank. An additional 72 loci potentially under balancing selection were found with BayeScan, including 59 found between regions, 19 within LC and one within NM. No outliers were found within TC. The neutral dataset had 3502 loci, the directional selection dataset had 193 loci and the balancing selection dataset had 72 loci.

For the neutral dataset, the greatest change in BIC values occurred at *K =* 3*,* with BIC values continuing to decrease until *K* = 6 (electronic supplementary material, figure S1). For *K* = 3, the groups corresponded to the three regions (LC, TC and NM) and a number of loci were found to be fixed within each region (LC: 2384, 68%; TC: 866, 25%; NM: 1609, 46%). A biplot of the principal components separated the samples into the three regions with 40.13% of the variation explained by differences between New Mexico and Texas, and 21.45% explained by differences between all three regions (electronic supplementary material, figure S2). Hierarchical AMOVA revealed significant heterogeneity among the regions (*F*_CT_ = 0.54, *p* < 0.0001) and among sites within the region (*F*_SC_ = 0.17, *p* < 0.0001). All post hoc pairwise estimates of *F*_ST_ were significant after correction (electronic supplementary material, table S2) and estimates of *F*’_ST_ ranged from 0.56, between LC and TC, to greater than 0.76, between LC and NM (table 1).

**Table 1.** Estimated pairwise *F*_ST_ (below the diagonal) and *F*’_ST_ (above the diagonal) between the Leon Creek (LC), Toyah Creek (TC) and New Mexico (NM) regions.

	LC	TC	NM
LC	—	0.5571	0.7626
TC	0.4809	—	0.6588
NM	0.6829	0.5509	—

For the directional selection dataset, BIC values fell sharply to *K* = 3 before decreasing gradually to *K* = 8 (electronic supplementary material, figure S3). A biplot of the principal components showed clear separation among the regions with 81.68% of the variation explained by differences between New Mexico and Texas, and 15.46% explained by differences between all three regions (electronic supplementary material, figure S4). Hierarchical AMOVA revealed significant heterogeneity among the regions (*F*_CT_ = 0.97, *p* < 0.0001) and among geographic samples within regions (*F*_SC_ = 0.12, *p* < 0.0001). Post hoc pairwise estimates of *F*_ST_ were significant for 63 of 66 comparisons after correction (electronic supplementary material, table S3). With all non-significant values between sinkholes in NM or between the sinkholes and the creek. Outliers indicative of directional selection were spread across all 24 major linkage groups in the *Gambusia affinis* genome, with a mean of eight loci per linkage group and an average distance approximately 4 MB between them.

For the balancing selection dataset, BIC values sharply decreased at *K* = 3, followed by a gradual decrease to a low at *K* = 6 (electronic supplementary material, figure S5). A biplot of principal components showed the same clear separation of the three regions and while the variance along the two primary axes was less than in the neutral and directional data, the within group variance was more apparent (electronic supplementary material, figure S6). Hierarchical AMOVA revealed significant heterogeneity among the regions (*F*_CT_ = 0.24, *p* < 0.0001) and among geographic samples within regions (*F*_SC_ = 0.12, *p* < 0.0001). Post hoc pairwise estimates of *F*_ST_ were significant also for 64 of 66 comparisons after correction (electronic supplementary material, table S4). Sink37 was not found to be significantly different from HEAD, though the *F*_ST_ value was higher than the average between LC and NM. As with the directional outlier data, BC was found to not be significantly different from Sink31. Outliers indicative of balancing selection were spread across all 24 major linkage groups in the *Gambusia affinis* genome, with a mean of three loci per linkage group and an average distance approximately 6 MB between them.

For the neutral dataset, estimates of *H*_e_ and *A*_r_ (electronic supplementary material, table S5) were significantly different among the geographic samples (*p* < 2.2 × 10^−16^). The lowest estimates of within population diversity were all in LC, except for Sink27, and the highest estimates of within population diversity were in TC (SS and ES; electronic supplementary material, table S5). Point estimates of contemporary *N*_E_ ranged from 22 (PHL) to 9404 (BLNWR). Overall, the smallest point estimates were in TC and the largest were in NM (table 2).

**Table 2.** Estimates of contemporary effective population size (*N*e) using the linkage disequilibrium approach for each sampling location with a 0.02% minor allele frequency threshold and no missing data (1569 loci).

location	*n*	pt. est.	low	high
KGS	38	937.2	445.8	Inf
EU	23	306.2	155.4	4575.3
SS	45	257.6	166.5	536.7
ES	22	179.6	123.9	318.8
PL	12	22.2	8.9	8723.3
BC	23	5263.9	782.6	Inf
BLNWR	21	9404.1	558.2	Inf

### 3.2. Phylogenetic and genetic demographic analysis

The final dataset for phylogenetic analysis contained 51 individuals, representing seven species of *Gambusia* and four outgroup taxa, genotyped across 5989 loci. The best fit nucleotide substitution model was determined to be TVMe using BIC. Topologies were consistent across analyses and clades well supported throughout the resulting topology (ML bootstrap support >96%), excluding a few terminal nodes with moderate to low support (figure 1; electronic supplementary material, figure S7). *Gambusia nobilis* formed a well-supported clade, nested within the remaining species of *Gambusia*. Within *G. nobilis*, there were three distinct clades with 100% support values, corresponding to the three regions. Toyah Creek and LC formed a monophyletic group, with NM as the sister taxon to this clade. Net divergence between the three groups ranged from 1.9% (TC versus LC) to over 3% (3.2% LC versus NM; and 3.3% TC versus NM). Divergence within regions ranged from 0.16% to 0.68% (table 3). Toyah Creek had the largest gdi value (0.732) which is above the 0.7 species threshold while both LC and NM had values (0.505 and 0.603, respectively) between 0.2 and 0.7.

**Table 3.** Estimated evolutionary divergence (*p*-distance) between clades, including total divergence (above the diagonal), within-group divergence (along the diagonal) and net divergence (below the diagonal).

	*G. nobilis* (LC)	*G. nobilis* (TC)	*G. nobilis* (NM)	*G. clarkhubbsi*	*G. heterochir*	*G. geiseri*	*G. gagei*	*G. speciosa*	*G. affinis*
*G. nobilis* (LC)	***0.0016***	0.0260	0.0343	0.1743	0.2675	0.1712	0.2297	0.4019	0.2864
*G. nobilis* (TC)	0.0194	***0.0068***	0.0382	0.1209	0.2661	0.3376	0.2738	0.3931	0.4992
*G. nobilis* (NM)	0.0319	0.0325	***0.0033***	0.1209	0.2661	0.3376	0.2738	0.3988	0.4992
*G. clarkhubbsi*	0.1221	0.1119	0.1185	***0.0014***	0.1743	0.2400	0.2038	0.2791	0.3688
*G. heterochir*	0.2657	0.2549	0.2634	0.1726	***0.0020***	0.1712	0.2297	0.2122	0.2864
*G. geiseri*	0.3376	0.3232	0.3331	0.2364	0.1673	***0.0020***	0.2718	0.2912	0.3155
*G. gagei*	0.2753	0.2677	0.2720	0.2029	0.2285	0.2687	***0.0004***	0.2448	0.3155
*G. speciosa*	0.3791	0.3670	0.3751	0.2564	0.1892	0.2663	0.2226	***0.0440***	0.1244
*G. affinis*	0.4917	0.4814	0.4849	0.3555	0.2727	0.3561	0.3027	0.0897	***0.0254***

*Gambusia clarkhubbsi* was used as the outgroup in the demographic analysis. Removing all missing data resulted in 7890 SNPs across 1585 loci and 746 873 bp of sequencing for the 27 individuals included (*G. nobilis*, *n* = 20; *Gambusia clarkhubbsi*, *n* = 7). The ESS for parameter estimates ranged from 1498 to 66 540. All split times were found to be within the last 50 000 years, though the timing varied depending on the mutation rate and generation time (table 4; electronic supplementary material, table S6). The most recent split, between LC and TC, was within the time frame of the Wisconsin glacial retreat (1566–17 045 YBP), while the split between NM and the two Texas groups was estimated to be as far back as 36 039 YBP. Historical effective population sizes ranged from over 1 000 to less than 14 000.

**Table 4.** Historical demographic parameter point estimates from BPP, including historical long term effective population size (*N*_E_) and split times for each region; Leon Creek (LC), Toyah Creek (TC) and New Mexico (NM), with 95% low and high confidence intervals.

	low	point	high
historical N_E_ NM	2204	3639	7873
historical N_E_ TC	3596	6101	13 393
historical N_E_ LC	1189	1950	4221
split NM and TC+LC	3364	11 218	36 039
split TC and LC	1566	5139	17 045

### 3.3. Morphological differences

**Figure 2.**
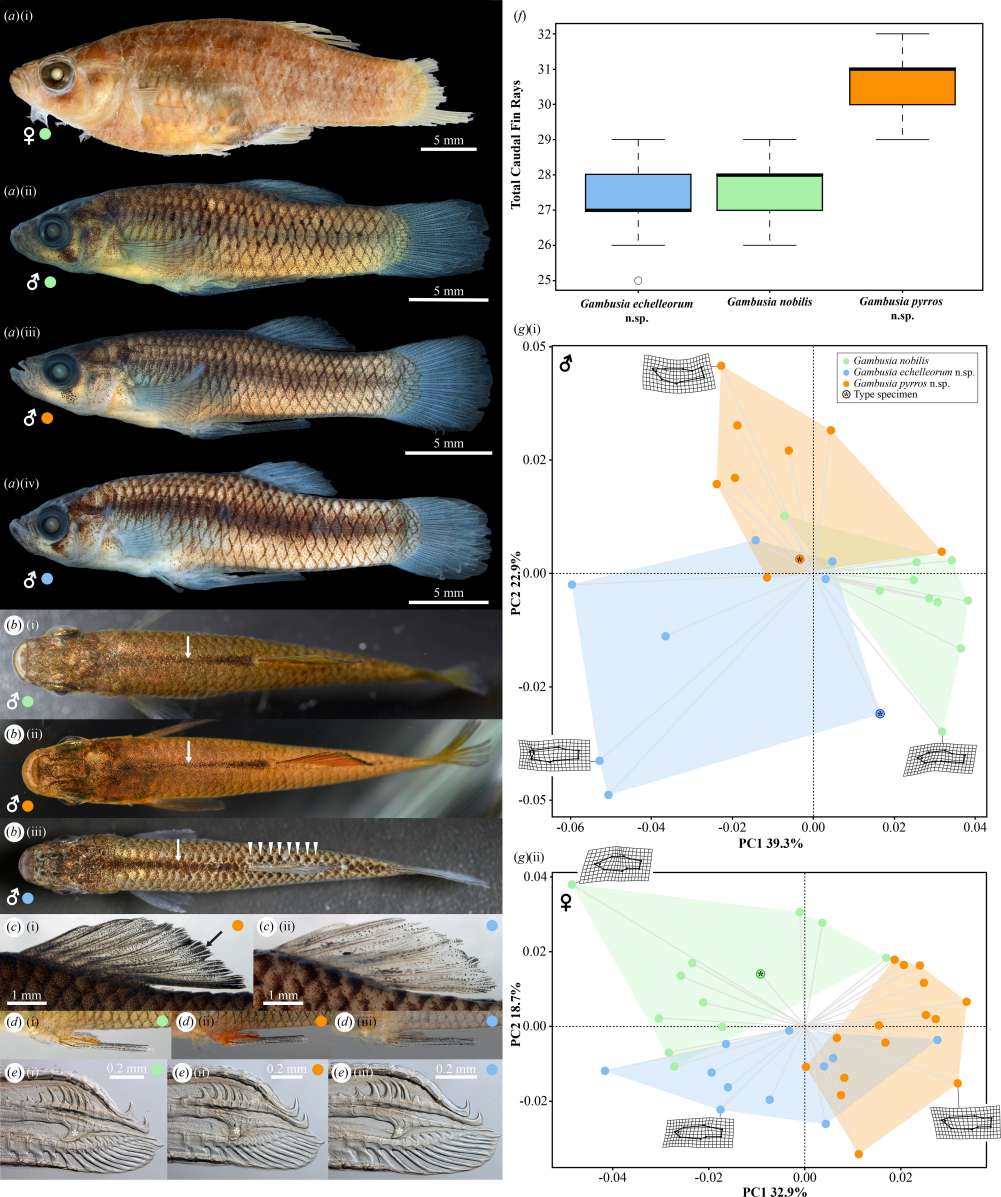
Morphological differences between members of the *Gambusia nobilis* complex, including *G. nobilis* (represented by green), *G. pyrros* n. sp. (orange) and *G. echelleorum* n. sp. (blue). (*a*) Specimens of the three species of *Gambusia*, left side (lateral view): (i) *G. nobilis*, female lectotype (MCZ 1455), 35.2 mm standard length (SL) (© President and Fellows of Harvard College); (ii) *G. nobilis*, male (TCWC 21102.01), 25.9 mm SL; (iii) *G. pyrros*, male holotype (TCWC 21103.01), 22.8 mm SL; (iv) *G. echelleorum*, male holotype (TCWC 21104.01), 24.5 mm SL. (*b*) Dorsal surface of male in life, white arrows points to middorsal stripe, white arrowheads point to blotches forming row adjacent to dorsal-fin base on right side of *G. echelleorum*: (i) *G. nobilis*; (ii) *G. pyrros*; (iii) *G. echelleorum*. (*c*) Dorsal fin (left side, lateral view) of preserved male holotypes, showing presence (black arrow) or absence of thick black band of pigment along distal margin of fin: (i) *G. pyrros* (TCWC 21103.01); (ii) *G. echelleorum* (TCWC 21104.01). (*d*) Gonopodium (left side, lateral view) of male in life: (i) *G. nobilis*; (ii) *G. pyrros*; (iii) *G. echelleorum*. (*e*) Distal tip of gonopodium in cleared and double stained specimen (left side in lateral view), elbow of ray 4 a highlighted by white asterisk (*): (i) *G. nobilis*, male (TCWC 21102.02); (ii) *G. pyrros*, male paratype (TCWC 21103.03); (iii) *G. echelleorum*, male paratype (TCWC 21105.02). (*f*) Box and whisker plot of total number of caudal-fin rays in *G. nobilis* (*n* = 25), *G. pyrros* (*n* = 24) and *G. echelleorum* (*n* = 28). (*g*) Scatter plots resulting from principal component analyses of the corrected landmark coordinates obtained from male and female specimens of the three groups, including the type specimen of each species represented by black asterisk (*): (i) male dataset; (ii) female dataset.

Notable differences in male colour pattern were identified among the three geographically isolated groups. Males from TC exhibit a bright orange-red or yellow-orange body colour in life (figures 1*d* and 2*b*(ii)), compared with a much duller yellow-brown (LC; figures 1*d* and 2*b*(i)) or yellow-grey to light cream (NM; figures 1*d* and 2*b*(iii)) (see also electronic supplementary material, figure S8). The base of the anal fin is bright orange-red in life in males from TC (figures 1*d* and 2*d*(ii)), compared with orange in males from LC and NM (figures 1*d* and 2*d*(i)(iii)). In males from NM a lateral stripe is well-developed in preserved material (figure 2*a*(iv)) and the middorsal stripe anterior to the dorsal fin is uniform in thickness (figure 2*b*(iii)), whereas in males from LC and TC the lateral stripe is absent or only weakly expressed after preservation (figure 2*a*(ii)(iii)) and the middorsal stripe is tapered anteriorly (figure 2*b*(i)(ii); see also electronic supplementary material, figure S8). A series of blotches formed by dense pigment located over the posterior part of scales adjacent to the base of the dorsal fin is another prominent feature of males from NM (figure 2*b*(iii)) that is absent in males from LC and TC (figure 2*b*(i)(ii)). Males from LC and TC exhibit a thick black band along the posterior edge of the dorsal fin (figure 2*c*(i)), which is absent (figure 2*c*(ii)) or only weakly expressed in males from NM (see also electronic supplementary material, figure S8). The colour pattern of preserved females is similar among the three groups. No difference in the number or configuration of gonopodial elements could be detected between the three groups (figure 2*e*). As reported earlier by Echelle & Echelle [19], we detected a significant difference in the total number of caudal-fin rays (one-way ANOVA, *F*[2,74] = 131.74, *p* < 0.001) and number of branched caudal-fin rays between genotyped individuals of the three groups (one-way ANOVA, *F*[2, 70] = 21.68, *p* < 0.001), with individuals from TC exhibiting higher numbers (figure 2*f*) than individuals from LC or NM (Tukey HSD test *p* < 0.001 for each comparison).

A total of 74.4% of the variation in male landmark coordinates and 64.8% of the variation in the female landmark coordinates was explained across the first three (PC1–3) of the 16 PC axes (see electronic supplementary material, PCA and PERMANOVA). Despite moderate overlap between individuals of the three groups in the PCA scatterplots for both the male (figure 2*g*(i)) and female dataset (figure 2*g*(ii)), a PERMANOVA of the PC scores (PC1–3) for each of the two datasets (male/female) revealed significant differences (*p* < 0.001) in body shape exist among the three groups (electronic supplementary material, table S7). This difference appears to be driven largely by individuals from TC in both the male and female datasets, though each of the six pairwise comparisons was found to be significant (*p* < 0.001; electronic supplementary material, table S7).

### 3.4. Taxonomy

Based on the evidence presented above, *Gambusia nobilis* (as currently defined) represents a complex of three geographically and genetically isolated species, two of which are currently undescribed. Below we provide a redescription of *G. nobilis*, which is restricted to the Leon Creek watershed, and descriptions of two new species (ZooBank registration: http://zoobank.org/661F5E15-17C7-43C9-B802-B1B82B876D0D).

#### 3.4.1. *Gambusia nobilis* (Baird & Girard 1853)

*Material examined:* All USA, Texas, Pecos Co. MCZ 1455, lectotype, female, 35.2 mm (figure 2*a*(i)); MCZ 1298, paralectotypes, 3 females, 27.5–37.0 mm SL; Leon Spring; coll: J.H Clark, 1851. TNHC 39733, 16 (not genotyped), 14 males, 18.7–27.8 mm SL, 2 females, 40.4–42.9 mm SL; Diamond Y Spring; coll: A. Echelle & C. Hubbs, 20 September 1972. TCWC 21102.01, 19 (genotyped), 9 males (figure 2*a*(ii)), 21.7–26.7 mm, 10 females, 24.8–36.5 mm; TCWC 21102.02, 6 (genotyped; c&s) 3 males, 21.7–22.4 mm SL, 3 females, 27.1–40.3 mm SL; Karges Spring (31°00′12.3″ N, 102°55′21.6″ W); coll: M. Bean *et al.*, 25 February 2024.

*Diagnosis:* A member of the *Gambusia nobilis* species group (*sensu* Rauchenberger [61]) most similar to *G. echelleorum* n. sp. and *G. pyrros* n. sp. *Gambusia nobilis* is distinguished from *G. pyrros* by: body colour of male yellow-brown in life (figures 1*d* and 2*b*(i); electronic supplementary material, figure S8) (versus orange-red or yellow-orange; figures 1*d* and 2*b*(ii); electronic supplementary material, figure S8), anal fin of male orange at base in life (figure 2*d*(i)) (versus orange-red; figure 2*d*(ii)), a lower modal number of total caudal-fin rays (26–29, mode 28 versus 29–32, mode 31; figure 2*f*), a lower modal number of branched caudal-fin rays (10–14, mode 12 versus 12–15, mode 14). *Gambusia nobilis* is distinguished from *G. echelleorum* by: body colour of male yellow-brown in life (figures 1*d* and 2*b*(i); electronic supplementary material, figure S8) (versus yellow-grey or light cream; figures 1*d* and 2*b*(iii); electronic supplementary material, figure S8), a higher modal number of pelvic-fin rays (6 versus 5), a poorly developed (figure 2*a*(ii)) (versus well-developed; figure 2*a*(iv)) lateral stripe along body side of male in preservative, scales on dorsal surface of male with sparse pigment (versus scales on dorsal surface of male with dense dark brown pigment on posterior part, forming short row of 7–8 dark brown blotches along dorsolateral surface of body adjacent to dorsal-fin base; figure 2*b*(iii)), middorsal stripe of male gradually increases in thickness towards posterior (figure 2*b*(i)) (versus uniform in thickness along length; figure 2*b*(iii)), dorsal fin of male edged with thick black band distally (figure 2*c*(i)) (versus uniform scatter of pigment cells across surface of fin or faint black band distally (figure 2*c*(ii)).

*Description:* Condition in female lectotype (figure 2*a*(i)), if available, denoted by asterisk (*). Body measurements and select counts are provided in electronic supplementary material, table S8 and a supplementary data file (measurements and counts). Gonopodial characters: ray 3 spines 10–11; segments contributing to elbow of ray 4 a 4; segments distal to elbow of ray 4 a 6–7; segments distal to ray 4 p serrae 5–6; ray 4 p serrae 5–6 (figure 2*e*(i)). Gonopodial suspensorium as described by Hubbs & Springer [18]. Dorsal-fin rays 8 (iii,4,i, ii,5,i or ii,6), 9* (ii,6,i or ii,7) or 10 (ii,6,ii or ii,7,i) (mode 9); anal-fin rays 9 (iii,6), 10* (iii,6,i) or 11 (iii,7,i) (mode 10); pectoral-fin rays 13 (iv,7,ii) or 14 (iv,7,iii) (mode 14); pelvic-fin rays 5 (i,3,i) or 6 (i,4,i) (mode 6); total caudal-fin rays 26–29 (mode 28*); branched caudal-fin rays 10–14 (mode 12); lateral scales 29–31 (mode 31); scales around caudal peduncle 14–17 (mode 16); pre-dorsal scales 15–17 (mode 15); vertebrae 31–32 (mode 32*). Largest male examined 27.3 mm SL, largest female examined 42.9 mm SL (both TNHC 39733; not genotyped).

*Coloration:* Colour of body in life yellowish-brown in both sexes, with iridescent blue sheen (figure 1*d*; electronic supplementary material, figure S8). Scale pockets edged with dark brown pigment, forming strong reticulate pattern. Flank of male peppered with small dark brown to black spots, most prominent on anterior half of body. Middorsal stripe of male dark brown, increasing in thickness towards posterior (figure 2*b*(i)). Dorsal fin faint yellowish-orange with black distal margin in both sexes. Caudal fin faint yellow at centre with dark grey distal margin in both sexes. Anal fin of female hyaline with dark grey distal margin, anal fin of male orange at base, with scattered black melanophores along shaft of gonopodium (figure 2*d*(i)). Base of anal fin in female bordered by black pigment, forming thick black band. A black suborbital bar below eye in both sexes.

*Distribution:* Springs and spring-fed tributaries of Leon Creek watershed within Pecos Co., Texas (figure 1*a*,*c*), including Leon (type locality [likely extirpated]), Diamond-Y, Karges and Euphrasia springs.

*Taxonomic remarks:* The redescription of *G. nobilis* provided by Hubbs & Springer [18] is based on material of both *G. nobilis* and *G. pyrros*, but not *G. echelleorum*.

#### 3.4.2. *Gambusia pyrros* new species

http://zoobank.org/DD777AE3-6253-4976-A765-80FAAB17D0C8

*Holotype:* TCWC 21103.01, male, 22.8 mm (figure 2*a*(iii)); USA, Texas, Reeves Co., outflow of San Solomon Spring within Balmorhea State Park (30°56′41.5″ N, 103°47′12.0″ W); coll: M. Bean *et al.*, 25 February 2023.

*Paratypes:* TCWC 21103.02, 17 (genotyped) 5 males, 20.9–26.4 mm, 12 females, 30.0–35.2 mm; TCWC 21103.03, 6 (genotyped; c&s), 3 males, 22.5–26.4 mm SL, 3 females, 28.8–33.7 mm SL; same data as holotype.

*Diagnosis:* A member of the *Gambusia nobilis* species group (*sensu* Rauchenberger [61]) most similar to *G. nobilis* and *G. echelleorum*. The characters distinguishing *G. pyrros* from *G. nobilis* are listed in the diagnosis of the latter. *Gambusia pyrros* is distinguished from *G. echelleorum* by the same characters that distinguish *G. nobilis* from *G. echelleorum*, plus: body colour of males orange-red or yellow-orange (figures 1*d* and 2*b*(ii); electronic supplementary material, figure S8) (versus yellow-grey to light cream; figures 1*d* and 2*b*(iii); electronic supplementary material, figure S8), anal fin of male orange-red at base in life (figure 2*d*(ii)) (versus orange; figure 2*d*(iii)), a higher modal number of total caudal-fin rays (29–32, mode 31 versus 25–29, mode 27; figure 2*e*), a higher modal number of branched caudal-fin rays (12–15, mode 14 versus 9–14, mode 12).

*Description:* Condition in male holotype (figure 2*a*(iii)), if available, denoted by asterisk (*). Body measurements and select counts are provided in electronic supplementary material, table S8 and a supplementary data file (measurements and counts). As for *G. nobilis*, with following differences. Segments contributing to elbow of ray 4 a 4–5; ray 4 p serrae 5 (figure 2*e*(ii)). Dorsal-fin rays 9* (ii,6,i or ii,7*) or 10 (iii,6,i or ii,8) (mode 9); anal-fin rays 10 (iii,6,i* or iii,7); pectoral-fin rays 13 (iv,7,ii or iii,8,ii), 14 (iv,6,iv) or 15* (iv,8,iii* or iv,9,ii) (mode 15); total caudal-fin rays 29–32 (mode 31*); branched caudal-fin rays 12–15 (mode 14*); scales around caudal peduncle 16; vertebrae 31–33. Largest male examined 41.2 mm SL, largest female examined 48.0 mm SL (both TNHC 7163; not genotyped).

*Coloration:* As for *G. nobilis*, with following differences. In life, body colour of male bright orange-red or yellow-orange, with greenish-blue iridescent sheen (figures 1*d* and 2*b*(ii); electronic supplementary material, figure S8). Dorsal fin bright yellow-red with black distal margin in both sexes, fin colour and black distal margin better developed in male. Lower caudal fin of male bright orange-red. Anal fin of male orange-red at base, shaft of gonopodium black (figure 2*d*(ii)). Pelvic fin of male reddish-orange, hyaline in female.

*Etymology:* From the Greek *pyrros*, meaning flame-coloured, a reference to the bright yellow, orange and red colours of the median fins of males in life. A noun in apposition. Proposed common name: flame gambusia.

*Distribution:* Springs and spring-fed tributaries of Toyah Creek watershed, including those in Reeves Co. (San Solomon [type locality], Giffin, and East Sandia springs) and Jeff Davis Co. (Phantom Lake Spring [likely extirpated]), Texas, USA (figure 1*a*,*c*).

*Taxonomic remarks:* Males collected from the type locality in 2024 (including holotype and paratypes) exhibited an orange-red body colour in life (see figure 1*d*; electronic supplementary material, figure S8). Underwater photographs obtained from the type locality in 2022 [62] and video obtained by the authors in 2023 [63] also document males with a bright yellow-orange body colour, which were considered a ‘rare male morph’ of *G. nobilis* by Echelle & Echelle [19]. Additional work is needed to characterize the range of life colours exhibited by males of *G. pyrros* and to further explore the potential drivers of this variation. See electronic supplementary material, extended methods for list of additional material examined.

#### 3.4.3. *Gambusia echelleorum* new species

http://zoobank.org/19CB8944-2C43-4180-AB49-525B0C0A8E84

*Holotype:* TCWC 21104.01, male, 24.5 mm (figure 2*a*(iv)); USA, New Mexico, Chaves Co, outflow drainage of Bitter Lake within Bitter Lake National Wildlife Refuge (33°27′36.1″ N, 104°24′08.3″ W); coll: M. Bean *et al.*, 26 February 2023.

*Paratypes:* All New Mexico, Chaves Co. TCWC 21104.02, 8 (genotyped), 1 male, 24.8 mm, 7 females, 29.7–36.5 mm; same data as holotype. TCWC 21105.01, 12 (genotyped), 8 males, 21.8–24.9 mm, 4 females, 28.7–32.3 mm; TCWC 21105.02, 7 (genotyped; c&s), 3 males, 22.5–24.8 mm SL, 4 females, 29.7–31.4 mm SL; Bitter Creek within Bitter Lake National Wildlife Refuge (33°28'38.2"N, 104°25'35.5"W); coll: M. Bean *et al.*, 26 February 2023.

*Diagnosis:* A member of the *Gambusia nobilis* species group (sensu Rauchenberger [61]) most similar to *G. nobilis* and *G. pyrros*. The characters distinguishing *G. echelleorum* from *G. nobilis* and *G. pyrros* are listed in the diagnoses provided for the latter two.

*Description:* Condition in male holotype (figure 2*a*(iv)), if available, denoted by asterisk (*). Body measurements and select counts are provided in electronic supplementary material, table S8 and a supplementary data file (measurements and counts). As for *G. nobilis*, with following differences. Ray 3 spines 9–10; segments contributing to elbow of ray 4a 3–4; segments distal to elbow of ray 4a 6; ray 4p serrae 5 (figure 2*e*(iii)). Dorsal-fin rays 8 (ii,6*) or 9 (ii,6,i or ii,7) (mode 8); anal-fin rays 10 (iii,6,i or iii,7); pectoral-fin rays 13 (iv,6,iii) or 14 (iv,7,iii) (mode 14); pelvic-fin rays 5 (i,3,i) or 6 (i,4,i) (mode 5); total caudal-fin rays 25–29 (28*, mode 27); branched caudal-fin rays 10–15 (mode 12*); lateral scales 29–31* (mode 30); scales around caudal peduncle 15–16* (mode 16); pre-dorsal scales 14*–16 (mode 15); vertebrae 31–33. Largest male examined 28.4 mm SL (CU 84258; not genotyped), largest female examined 36.5 mm SL (TCWC 21104.02).

*Coloration:* As for *G. nobilis*, with following differences. Colour of body in life yellow-grey to light cream in both sexes, with iridescent blue sheen (figure 1*d*; electronic supplementary material, figure S8). Horizontal dark brown to black lateral stripe along body side of male in preservative (figure 2*a*(iv)), formed by pigment cells located on scales in midlateral scale row and lower or upper half of scales in row directly above or below midlateral scale row, respectively. Middorsal stripe dark brown, equal in thickness along length (figure 2*b*(iii)). Scales along base of dorsal fin with dense dark brown to black pigment over posterior part, forming horizontal row of 7–8 dark brown blotches along dorsolateral surface of body adjacent to dorsal-fin base (figure 2*b*(iii)). Pigment cells uniformly scattered across surface of dorsal fin or concentrated along distal margin, forming weak grey or black band (figure 2*d*(ii)).

*Etymology:* Named for Alice and Anthony Echelle in honour of their work on *Gambusia nobilis*. A noun in the genitive. Proposed common name: New Mexico Gambusia.

*Distribution:* Spring fed creeks and sink holes within Bitter Lake National Wildlife Refuge, Chaves Co., New Mexico (figure 1*a*,*b*).

*Taxonomic remarks:* Based on information available from Echelle *et al.* [20] we expect (but have not been able to confirm) that the population of *Gambusia* in Blue Spring (Eddy Co., NM) belongs to *G. echelleorum*. See electronic supplementary material, extended methods for list of additional material examined.

## 4. Discussion

*Gambusia nobilis* is a federally endangered species with a fragmented distribution across the Pecos River system [19], a situation that would be expected to result in population structuring (as suggested by allozyme-based analysis [20]), especially given small population sizes and short generation times. The levels of genome-wide differentiation detected in this study between regions, however, were much greater than expected with estimates of pairwise *F*_ST_ as much as 76% of the maximum possible value. Subsequent phylogenetic analysis revealed that the regions formed reciprocally monophyletic groups that together represent the sister taxon of *G. clarkhubbsi*, a species also found in the Rio Grande drainage [64]. Though estimates of genome wide divergence between the three geographically isolated groups were low (1.9%–3.3%) relative to other species comparisons within *Gambusia* (range 9.0%–49.2%; table 4), the average net divergence among the three groups (2.8%) is nearly an order of magnitude greater (approx. 7X) than the average divergence within regions (0.39%), consistent with the idea that *G. nobilis* is a species complex [65,66]. Furthermore, gdi values were statistically greater than 0.2 indicating divergence beyond what is expected within a species, with point estimates for TC consistent with species (0.7), and those obtained for LC and NM in the zone of indecision [67] (0.5 and 0.6, respectively), consistent with values reported for subspecies or allopatric species [68]. Subsequent morphological analyses also supported significant differences among the three geographic groups and identified differences in phenotypic characters, including aspects of male colour pattern and fin-ray counts (in both sexes). Because of potential admixture with introduced congeners, this study relied on contemporary samples that could be genetically confirmed as ‘pure’ and this somewhat limited sample size for morphological analyses because of the species Endangered status. None-the-less, based on the total evidence, *G. nobilis* should be considered three species rather than one, and henceforth the name *G. nobilis* should be applied only to individuals found within the Leon Creek Basin of Texas. Individuals found further to the west in Texas in the Toyah Creek Basin represent *G. pyrros,* and those individuals that occur within the Bitter Lake System of New Mexico represent *G. echelleorum*.

While directional selection is often implicated as an important driver of speciation [11,69], the results of this study suggest genetic drift may have been more important for creating observed patterns of divergence. The primary component of structure corresponds to species-level differences not only in the neutral dataset, but also in the outlier datasets, with substructure present within each species (electronic supplementary material, figures S2, S4 and S6). While outlier tests are meant to detect regions of the genome influenced by selection [35], outliers can also represent regions of the genome where allele frequency changes mediated by genetic drift are lower or higher than expected (i.e. drift outliers [70]) and corresponding patterns across datasets suggests that this maybe the case. Islands of divergence (genomic regions of elevated divergence) are often used as evidence of localized selection promoting speciation [71,72], but none were detected as outlier loci appear evenly distributed within and across chromosomes. The time it takes for lineages to sort via drift is dependent on levels of recurrent gene flow among diverging gene pools and the effective size of those gene pools [73,74]. The complete geographic isolation of the three species of the *Gambusia nobilis* species complex (*G. nobilis*, *G. echelleorum* and *G. pyrros*) and small effective size estimates indicate that drift could efficiently cause allele frequencies to diverge, a process that is estimated here to have occurred over tens of thousands of generations (table 4). The estimated divergence times reported here (<50 000 years before present) correspond to climatic fluctuations during the mid to late Pleistocene [75,76], rather than Pliocene monsoons previously implicated as drivers of biodiversity patterns in the region [15]. These results are similar to those reported in a recent study on the white sands pupfish species complex [77], and like that example, environmental differences among spring fed habitats are not likely great enough for selection to overcome genetic drift operating on small, isolated populations.

It is important to note that within *Gambusia echelleorum* there were some small and non-significant pairwise *F*_ST_ estimates for the directional selection dataset, and all of those comparisons involved populations in sinkholes (electronic supplementary material, table S3), perhaps signifying adaptive differences between sinkhole and creek run populations. However, the non-equilibrium population dynamics of small, isolated populations such as these tend to violate the assumptions of standard genomic outlier tests [78] obscuring patterns of selection and future investigation using moderate coverage whole genome shotgun sequencing could provide further evidence for selection.

Desert ecosystems are extreme environments where water scarcity presents a major challenge to flora and fauna, potentially limiting biodiversity. Because these arid regions are proximal to areas which are less water limited, they are often inhabited by taxa found more broadly but that are preadapted to living in harsh conditions [79]. Furthermore, suitable habitats for specific species in arid regions (e.g. the southwestern United States) are often fragmented, especially in the case of aquatic species [13,80]. Biogeographic analyses of desert systems have documented the importance of periodic founder events for seeding new populations for species with fragmented distributions [81]. For aquatic species in the southwestern United States, periods of increased precipitation during climatic cycles of the Pleistocene and immediately afterwards [16] created more extensive waterways, connecting regions that are now completely geographically isolated. Results of demographic analyses in this study are consistent with such a dynamic, but with isolation occurring during or just after the last glacial period. A series of climatic shifts promoting separate incidents of peripatric speciation would lead to groups of endemic species each occupying a small range, that diverged over a range of times, a pattern seen in a variety of other southwestern United States taxa (e.g. *Cyprinodon*, *Dionda*, *Pyrgulopsis*, *Gammarus* [15,82,83]), that contrasts greatly with concordance resulting from singular vicariance events [84]. Because of the importance of drift relative to selection in small, isolated populations and the general similarity among the spring fed habitats in which these species are found, widespread morphotaxa in desert ecosystems may harbour undescribed species diversity [12], as exemplified herein. Taken more broadly, this study and other recent studies [77,85,86] suggest that species diversity in aquatic environments in arid bioregions is underestimated. Given the imperilled nature of these habitats and the species within them, due to climate change, water usage and other anthropogenic forcers, there is significant risk for biodiversity loss before it is even recognized.

Species delimitation has become a contentious topic in recent decades [87] as new technologies and approaches have enabled finer scale assessments of biodiversity than was previously possible [6]. During a time of unprecedented risk for loss of diversity, properly identifying species is more than a semantic discussion, as properly recognized species may need protections otherwise not afforded to them [88]. Alternatively, the incorrect division of a single species into multiple species can create financial and societal inefficiencies [89,90] and may doom small populations to extirpation by limiting the potential for genetic rescue [91]. In this context, the decision to describe new species should involve careful consideration of species concepts and associated criterion used to distinguish differences that support species recognition [92]. Speciation is a dynamic process and the relative differences between any two species lies on a continuum [93], making it difficult to define a ‘one-size-fits-all,’ rule for delimitation. For example, evidence of reproductive isolation (e.g. outbreeding depression, inability to reproduce) could be considered robust evidence of speciation in a conservation context [91]. But freshwater fishes readily hybridize [94], including *Gambusia*, and in some cases with evidence of hybrid vigour [95,96]. Post hoc analyses show that for approximately 14% of the loci present (525/3770) one of the species does not share allelic variation with at least one of the other species, a potential indication of outbreeding depression [91], but precluding the ability to perform in multigeneration breeding experiments to confirm outbreeding depression, the proposed criterion are impractical to deal with. Galtier [93] suggests a reference-based approach in which levels of divergence among putative species are compared to those among well recognized species, but such an approach may fail to detect cryptic species, which are often recently diverged and thus morphologically similar. The point of the last two examples is not to deride the ideas presented by the authors but to demonstrate the difficulty of developing a unified system of species delimitation that fits in all cases.

Here a total evidence approach was applied, in which both genetic and morphological data were assessed. At the heart of such an approach, is the consideration of whether multiple data sources (genetic and morphological) produce congruent patterns that support species delimitation [97]. The evolutionary species concept defines a species as a ‘lineage … which maintains its identity from other such lineages and has its own evolutionary tendencies and historical fate’ [98] and in the case presented here, analyses support the evolutionary independence of the three proposed species. Though recently diverged, the species are reciprocally monophyletic with patterns of variation at a genome-wide scale indicating rapid divergence and complete cessation of gene flow. Furthermore, both males and females show identifiable (though subtle) gross morphological differences consistent with the observed genetic differences. Given current and future climatic conditions in the southwestern United States, it is unlikely that the three species will come back into contact in the wild, unless the contact is mediated by humans, and thus will continue to move apart, ensuring decoupled fates.

## Data Availability

Raw HiSeq reads are available in the NCBI SRA (https://www.ncbi.nlm.nih.gov/bioproject/PRJNA1337111). Analysis scripts, genetic and morphological data are available on GitHub [99] and Dryad [100]. Sample metadata are available in electronic supplementary material, data file 1 [101].
